# Clinical Instruction at the Philadelphia Hospital

**Published:** 1860-11

**Authors:** 


CLINICAL INSTRUCTION AT THE PHILADELPHIA HOSPITAL.—By the liberality of the Board of Guardians, the wards of this extensive charity have been thrown open to students, for the purpose of clinical instruction, free of charge. Clinical lectures have already been commenced, Drs. Ludlow, Agnew, and Penrose being on duty. We advise all students to avail themselves of the varied and extended opportunities now gratuitously offered to them. The lectures are delivered in the amphitheatre every Wednesday and Saturday, beginning at ten o'clock A.M.

